# Development of an educational and monitoring mobile application for pregnant women in Nigeria

**DOI:** 10.3389/fpubh.2024.1368631

**Published:** 2025-01-22

**Authors:** Abdulhammed Opeyemi Babatunde, Adejumoke Idowu Ayede, Amalia Colangelo, Tuan Dung Nguyen, Abdullahi Tunde Aborode, Charles Umeh, Maria Paula Hernandez, Oluwaseyi Iyanuoluwa Ayede, Oluwatobiloba Oluwadunni Ayede

**Affiliations:** ^1^College of Medicine, University of Ibadan and University College Hospital, Ibadan, Oyo State, Nigeria; ^2^MyBelle Digital Maternal and Child Health Organisation, Ibadan, Nigeria; ^3^Centre for African Newborn Health and Nutrition, University College Hospital, Ibadan, Oyo State, Nigeria; ^4^Benjamin S. Carson College of Health and Medical Sciences, Babcock University, Ilishan-Remo, Ogun State, Nigeria

**Keywords:** mHealth, mobile application, pregnancy, educational, monitoring

## Abstract

**Introduction:**

Nigeria accounts for 20% of all maternal mortality. Recently, more mobile health technology (mHealth) interventions are emerging in sub-Saharan Africa. The potential of mobile applications in maternal care has not been explored in Nigeria. This study describes the process of design, development, and testing of an educational and monitoring mobile application for pregnant women in Nigeria.

**Method:**

Using a user-centered design, we conducted semi-structured interviews at each stage of mobile application development with pregnant women attending antenatal clinics in Oyo State, Nigeria. The first interview focused on empathy, followed by alpha and beta testing of the mobile application prototype at health facilities.

**Results:**

The barriers to accessing perinatal care were the distance to the nearest facility (mean = 3.3 km), lack of perinatal education, and cost. The low-fidelity prototype of the mobile application was designed with five features. Mobile applications increased the level of knowledge of preeclampsia by 179%. User feedback from alpha testing informed the development of a high-fidelity prototype for beta testing. Ninety-five percent (95%) of pregnant women surveyed were willing to download the mobile application. The final application developed was uploaded to the Google Play Store (MyBelle pregnancy application).

**Conclusion:**

mHealth applications have the potential to increase access to prenatal information and services in Nigeria and may reduce maternal and childhood mortality. This study has described the process of development of the first indigenous mobile application specifically for pregnant women in Nigeria using a user-centered design thinking approach.

## Introduction

Maternal and childhood mortality are public health issues disproportionately affecting low- and middle-income countries. Approximately 99% of global maternal mortality occurs in developing countries, with sub-Saharan Africa accounting for two-thirds of the global burden (1). Although significant progress was recorded through the Millennium Development Goals (MDGs) to reduce child mortality and improve maternal health by 2015, approximately 80% of sub-Saharan African countries were not on track to meet both MDGs 4 and 5 (2). A similar trend has been described for the Sustainable Development Goal for 2030. The average maternal mortality rate in sub-Saharan Africa is approximately 533 maternal deaths per 100,000 live births with countries such as South Sudan, Chad, and Nigeria having rates greater than 1,000 (1, 3). Nigeria has the highest maternal mortality after India with a rate of 1,047 deaths per 100,000 live births in 2020 (4). The rate of reduction of maternal mortality in Sub-Saharan African countries and other developing countries is still low to attain the SDG by 2030. Political instability, insurgencies, economic crises, and the COVID-19 pandemic are some notable factors affecting maternal and child mortality in developing countries. The major causes of maternal mortality in sub-Saharan Africa and globally are post-partum hemorrhage, hypertensive disorders in pregnancy (mainly preeclampsia and eclampsia), sepsis, and complications of delivery (5). However, poverty, distance to healthcare facilities, cultural practices and religious beliefs, lack of information, and inadequate or lack of quality health services have been described as major barriers to accessing maternal and child healthcare (1, 6).

In a systematic review conducted by Alghamdi et al. (7), mobile health (mHealth) technology was found to improve access to care in developing countries. mHealth technology has been used in developing nations for health education and promotion, disease self-management, decreasing long-term healthcare expenditure, and remote monitoring (7). The COVID-19 pandemic and high digital penetration in African countries have facilitated the adoption of mHealth in several healthcare areas in the form of short message services (SMS), calls, websites, and mobile applications. In addition, mHealth in maternal health was found to result in the most prominent health outcomes in developing countries compared to other areas of health (8). The majority of initiatives have used mainly SMS either alone or in combination with other mHealth interventions. However, mobile applications have been found to be effective in improving healthy lifestyles during pregnancy, antenatal visit adherence, and pregnancy outcomes in some low- and middle-income countries (LMICs) outside Africa (9, 10). Despite the potential of mobile applications in increasing access and improving maternal and child health in sub-Saharan Africa, it has not been maximally explored. This may be due to several barriers specific to mobile application usage in the sub-region, such as lack of infrastructure, poor digital literacy, poor Internet connectivity, and power supply (8). In recent times, mobile applications for pregnant women, nursing mothers, and healthcare workers including traditional birth attendants have been developed with different goals but with the ultimate aim to reduce maternal and childhood mortality (9, 11, 12). Some identified gaps in the available mobile applications include language barrier, lack of local context, high costs to access quality information and services, the requirement of high bandwidth, and a lack of specificity to health issues. A team of engineers, doctors, and scientists identified this gap and developed a mobile application (MyBelle pregnancy application) using the user-centered design thinking approach for pregnant women in Nigeria and other resource-limited countries or regions. Hence, this manuscript aims to (1) outline the process of design, development, and testing of the mobile application; (2) discuss the challenges and solutions identified; and (3) highlight key lessons learned for future mHealth applications for maternal and child health in sub-Saharan Africa.

### Features and functionality of the mobile application

The mobile application is designed with five key features to provide free information and services for pregnant women and nursing mothers. The features of the mobile application include (i) a dashboard, (ii) an educational platform, (iii) lifestyle tracking, (iv) a financial planning toolkit, and (v) access to the clinic. The launched application contained only three functioning features, namely, a dashboard, an educational platform, and lifestyle tracking. The application’s dashboard includes a week of gestation, estimated date of delivery (EDD), motivation, and profile including a reward system for interaction with users on the application. The mobile application provides educational content (both in English and local languages) and pregnancy tips tailored to the user’s estimated gestational age, along with quizzes. The lifestyle tracking features allow users to assess and keep a record of their mental health, mood, schedule/plan, blood glucose levels, blood pressure, and the number of times the baby kicks.

## Methods

### Study design, participants, and setting

The development of the mobile application utilized a mixed-method study design. Participants were pregnant women attending an antenatal clinic in Oyo State, Nigeria.

### Sampling strategy

Convenience sampling was used. Pregnant women who were interested in participating in the testing were included.

## The process of mobile application development

### Need assessment

We conducted a needs assessment survey by interviewing pregnant women in August 2020 at the Oyo State General Hospital in Oyo Town, Nigeria, to empathize with pregnant women in the community and understand their pain points. Our findings corroborated with online research. The proposed features of the mobile application were shared in the interview for rating and feedback. Afterward, we conducted a qualitative survey via the Internet using Google Forms to get responses from women of childbearing age.

The feedback and responses from pregnant women and an online survey for women of childbearing age informed the design of the high-fidelity prototype of the five features on Figma is a tool for designing interface for products www.figma.com. The prototype was assessed by pregnant women at the Oba Adeyemi Primary Health Care Centre, Oyo, Oyo State, Nigeria, on 5 January 2021.

### Prototype testing

**Figure 1:**
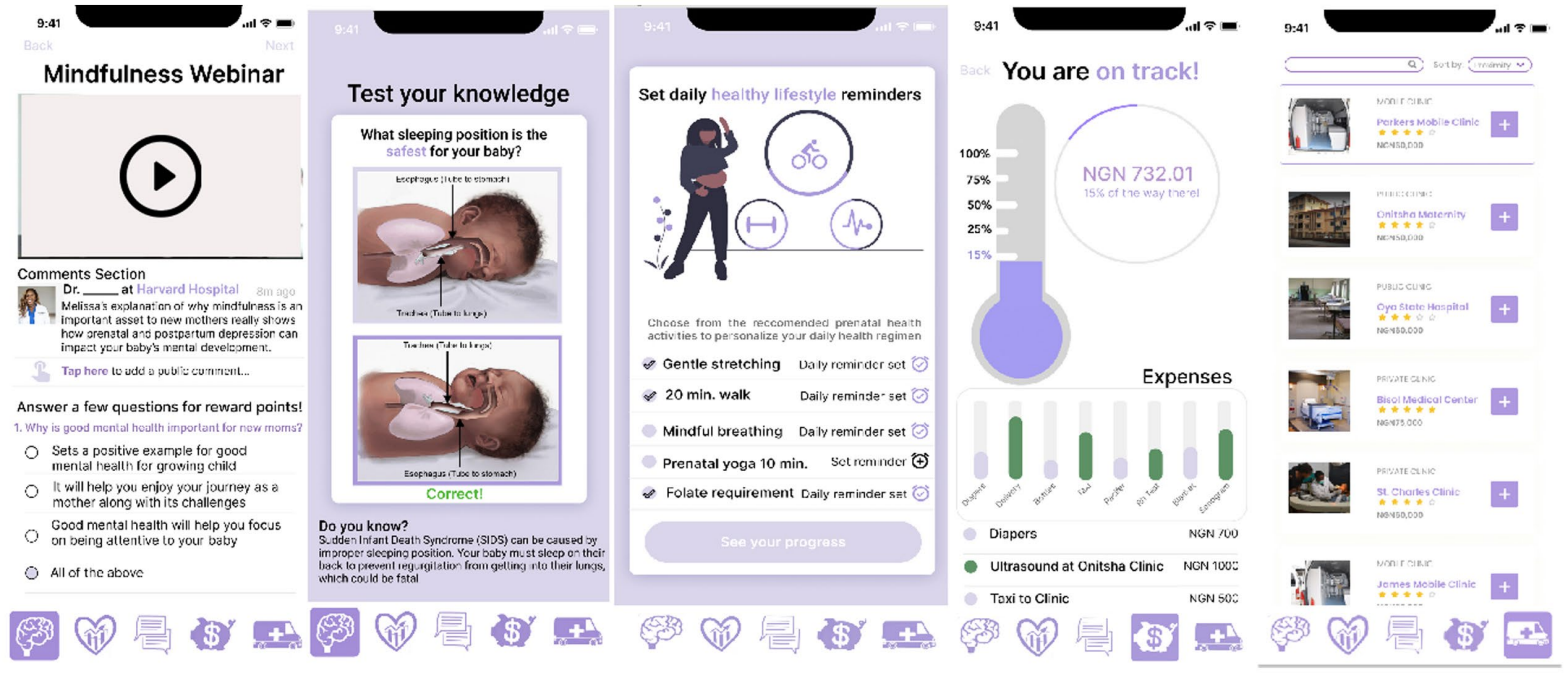
UI/UX screens of the MyBelle mobile application prototype.

A mixed-method study design was used for testing. This involved a self-administered questionnaire to collect quantitative data followed by a qualitative study through an interview with each tester. The low-fidelity prototype (Figure 1) of the mobile application was developed using React Native. We only included the gamified microlearning features, which are the most desired features of the application from the prospective user survey. It contained only articles and quizzes on preeclampsia and nutrition, which are among the major causes of maternal mortality in Nigeria (13). The alpha testing of the mobile application was conducted at the Oyo West Primary Health Care Centre, Iseke, Oyo. Each woman used the prototype for 30 min and was given a pre-survey and a post-survey.

High-fidelity prototype testing (beta testing) of the mobile application was carried out in June 2021 at the Adeoyo Maternity Teaching Hospital, Ibadan. This included the sign-up button, application dashboard, lifestyle tracking and recommendation, and gamified microlearning features. The Android file was shared with interested pregnant women at the clinic to install and interact with the application.

The final application developed included robust functions for each of the features. The profile page included the personal information of the user and user point rewards. The user dashboard is the first display upon opening and signing into the application. It includes the week of pregnancy, gestational age-specific health tips, motivation, and exercise.

The functionality of each of the five features is highlighted below: (1) Gamified microlearning: this feature allows users to access educational content in both English and local languages (in text and audio formats), along with games, quizzes, and pre-recorded videos from maternal and child health specialists. (2) Biometric tracking and lifestyle recommendations: this feature helps users apply the knowledge gained from the gamified microlearning to create personalized and actionable prenatal goals. It supports physical, mental, and emotional wellbeing through tools such as calendar reminders, mood trackers, kick counters, mental health assessments, and glucose and blood pressure recorders. (3) Clinic connection: this feature allows users to connect with nearby stationary and mobile clinics that offer prenatal care services to circumvent distance-induced barriers to receiving female reproductive healthcare. (4) Financial planning: this feature allows users to build a personalized saving plan based on the estimated cost after an informed decision in connecting to the clinic. The saving frequency can be set to daily, weekly, or monthly, with reminders. In addition to saving, this feature also allows women to input their expenditures throughout their pregnancy. (5) Chat option: this feature links users to WhatsApp to allow direct communication with healthcare professionals.

**Figure 2:**
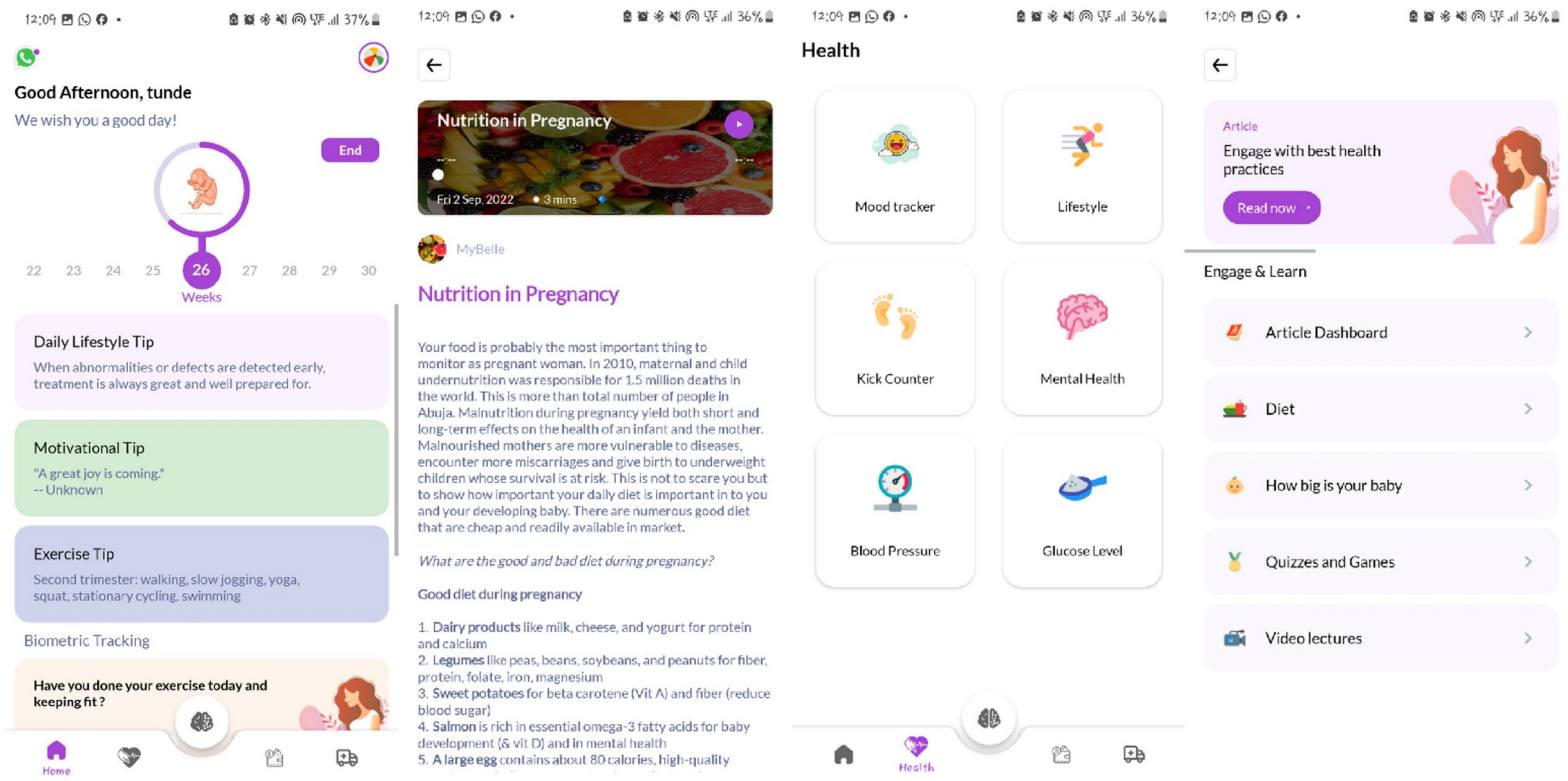
Screens of the MyBelle pregnancy application (final version).

The final release (MyBelle pregnancy application) was developed using the Android Studio environment and the Kotlin programming language (Figure 2). The application works on devices running the Android operating system version 7.0 to 12. The mobile application was developed in the English language which is most widely spoken in the targeted geographical area. An administrator application was also developed for real-time uploading of contents, which include articles, quizzes, diet plans, clinics, and videos by the managing team. The application coding lasted for approximately 8 weeks. The functionality and performance of the application were tested on several runs. During each test run, the application was installed on an Android device by a researcher, product manager, and developer individually. The bugs encountered during each test were corrected. The functions of each feature of the application including the onboarding process were evaluated for 2 to 3 weeks before being tested by prospective users at community outreaches to primary healthcare centers in Ibadan North Local Government. The APK file was finally uploaded to the Google Play Store for free download and launched online on 3 December 2022.

### Content development

The medical contents on the application in the form of articles, videos, mental health assessments, and weekly lifestyle tips were developed by the co-founders with medical backgrounds and reviewed by resident doctors and consultants from the University College Hospital, Nigeria. Sources of the contents include research publications, recognized health websites (such as the WHO, UNICEF, Healthline, NHS UK, and Baby Center), and medical textbooks. We have a diverse team of advisors with expertise in obstetrics and gynecology, pediatrics, public health, and AI in healthcare. The articles were simplified and concise for easy readability and comprehension by users with a maximum word count of 500 words each. The articles were written in English and translated into popular local languages (Yoruba, Hausa, Igbo, and Pidgin) after being reviewed. The audio recordings of the texts are attached to the articles being uploaded. In addition, the mobile application was developed to provide an opportunity for real-time uploading and updating of content on the mobile application from the backend.

**Figure 3:**
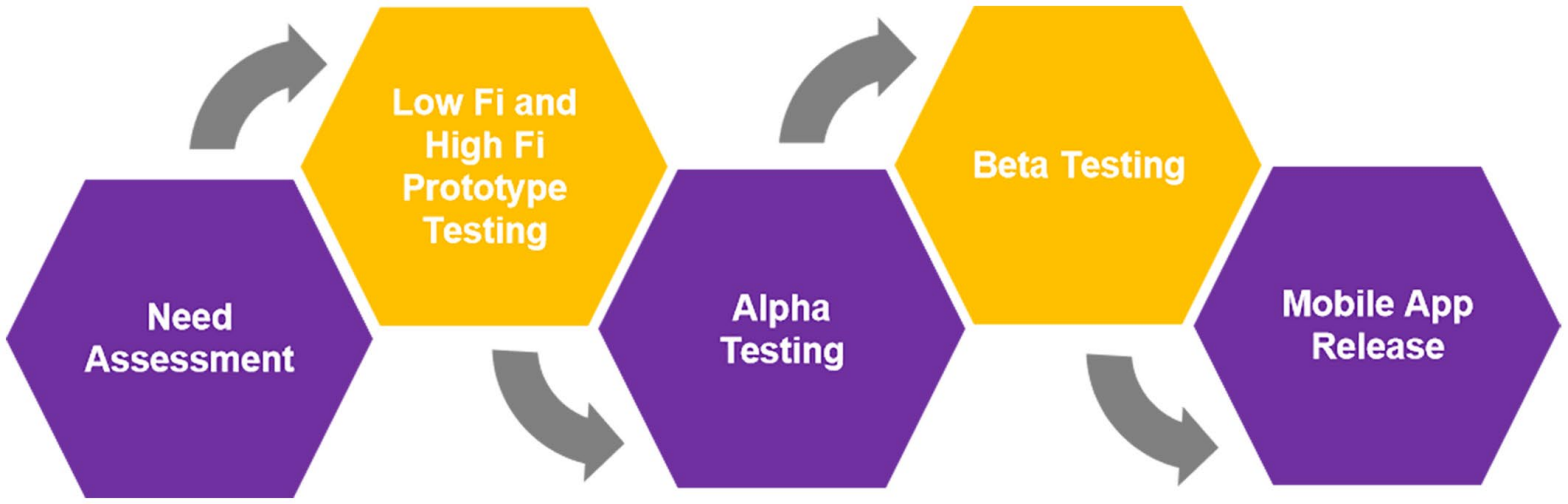
MyBelle mobile application development process.

Figure 3 summarizes the process of the mobile application development.

### Data collection instruments

The qualitative data were collected through in-depth interviews. The interview prompts were designed by the research team based on the aim of each phase. The quantitative data were collected with a questionnaire. The first draft of the questionnaire was developed through a review of published research on preeclampsia and maternal health. This was revised by experts in obstetrics and gynecology and public health and tested among women of reproductive age group.

### Data analysis

Quantitative data collected were input into Google Sheets, cleaned, and organized. Descriptive statistics was conducted to determine the frequency, mean, and percentage. The data were presented in the form of tables and figures. The qualitative data were transcribed, and thematic analysis was conducted to identify the key and recurring needs of pregnant women.

### Ethical considerations

The study was conducted during the COVID-19 lockdown, and no formal ethical approval was collected from the institution’s ethical review body. However, we strictly adhered to all ethical issues involved in conducting studies involving human participants, such as obtaining informed consent, which clearly stated participants’ right to withdraw from the study at any time. In addition, we ensured confidentiality and anonymity by not requesting any personal or identifiable information on the survey. Anonymized data were stored on the organization’s drive and encrypted for privacy.

## Results

### Need assessment

A total of 25 pregnant women were interviewed. Major challenges faced by the women included distance to healthcare facilities (average of 3.3 km) and additional cost of antenatal and delivery materials. All the women owned a smartphone, but only four had access to the Internet, with an average monthly Internet cost of NGN1,175 ($2.9). The women surveyed lived an average of 3.31 km away from the health facility. Seventeen (68%) believed they would have attended more antenatal visits if they lived closer. Lifestyle tracking (*n* = 7) and gamified prenatal microlearning (*n* = 7) were voted as the most useful features of the mobile application.

### Testing

A total of nine pregnant women at the health center were enrolled in the low-fidelity prototype testing (alpha testing). Approximately 78% of the women surveyed did not know about preeclampsia prior to the application experience, and an average of 179% increase in the knowledge of preeclampsia was recorded after interacting with our application. All women liked the MyBelle application experience and gave a few recommendations such as the need to chat with MyBelle, incorporation of audio content, use of local language, need for offline access, and EDD. Figures 1, 2 show the difference between the low-fidelity prototype and the final version launched based on the feedback.

A total of 24 pregnant women were enrolled in the high-fidelity prototype testing (beta testing). Approximately 95% of women surveyed were willing to download the MyBelle pregnancy application on their phones. The simplicity and concept of the User Interface (UI)/ User Experience (UX) were the top reasons why pregnant women desired the application.

## Discussion

The MyBelle pregnancy application is a holistic mobile application that addresses the common challenges of pregnant women in Nigeria and other developing countries. The mobile application design adopted the human-centered design thinking process, which enables co-creation of the application with the end users at every stage of the development. The mobile application is designed to influence the behavior and mindset of users to adhere to antenatal visits, promote a healthy lifestyle in pregnancy, monitor expenses, and improve access to healthcare facilities offering maternal and child services. The educational contents are short and precise to address pregnancy-related topics, such as nutrition, infection, mental wellbeing, preeclampsia, and gestational diabetes mellitus. This will increase the knowledge of pregnant women and reduce mortality. The lifestyle tracking features aid monitoring of users such as mental health, mood, blood glucose, and blood pressure, thereby facilitating early detection of physical or mental health challenges. In addition, it serves as a means of generating quality data to influence decision-making. The dashboard improves user experience by providing daily motivation and tips, as well as a link to the MyBelle WhatsApp Community. The functionality of the application is holistic, and the UI/UX design enables easy navigation with a maximum of three to four clicks to access any functionality on the application. In addition, users do not require the internet except during the onboarding process and the first time fetching content. This reduces the cost of the internet and increases its usability in areas with poor internet connectivity.

### Challenges and lessons learned

The application development was faced with some challenges and limitations. First, the need assessment and ideation of the mobile application occurred during the COVID-19 pandemic lockdown which limited access to pregnant women and healthcare facilities. In addition, the cultural and religious beliefs in the Oyo community discourage disclosure of personal problems especially to strangers, thereby making it difficult to understand the pain points of pregnant women surveyed. In addition, the process of seeking approval for user surveys at some of the healthcare facilities was challenging. Some participants during the testing also disclosed challenges in the sense of rating, which might have caused bias in the results of testing. There is poor documentation and a paucity of research publications on already existing mobile health applications in Nigeria and other African countries. The diversity of Nigerian populations is a major challenge such as differences in culture including values, norms, diet, and language, which may hinder the usability of the mobile application by every woman. For instance, many Northern Nigerian women do not have sole ownership of mobile devices, and this is not common in Southern Nigeria (14). Hence, there may be a need to adjust innovation to suit the custom. In addition, despite high digital penetration in the country, digital literacy remains low, even among educated populations. Other challenges such as poor infrastructures like stable power supply and Internet connectivity, financial constraints, and social and domestic issues have been described in a similar study (15). Some limitations of this study are that the current version of the application is only available for Android-enabled devices and in the English language. Providing the iOS version and other languages will be considered while developing the next version of the application. The number of pregnant women who tested the application at each stage was small although the size was sufficient to obtain reliable feedback. In the future, the number of participants in the testing stages should be larger.

### Sustainability and scalability of the mobile application

The long-term sustainability of the mobile application is important for achieving the desired impact. The application will be sustained through several streams such as research funding, government partnerships, and commissions from private health insurance for premiums purchased from the application, paid premium features, and advertisement for relevant products. The mobile application will be scaled to other regions in Nigeria from the South-West. The features will be scaled to include other services desired by pregnant and nursing mothers. In the long term, the mobile application will be scaled to other African countries with similar demographics as Nigeria.

### Limitations

Although this study described the process of developing a mobile application for pregnant women in Nigeria, there were some limitations. First, the sampling technique was non-randomized, which might have increased bias in the results. In addition, the sample size for each stage of development was small, which limits the generalizability of the findings. The need assessment and testing were focused on a single region in the country whereas the mobile application was developed for the entire country. This was due to the pandemic and lack of funding.

### Recommendations

We recommend that future innovations should also adopt human-centered design thinking to co-create the innovation with prospective users and stakeholders such as healthcare workers, community leaders, and partners. There is a need for collaboration with non-governmental organizations and healthcare providers in Nigeria to increase reach and impact. There is a need for funding and support from ministries and industry experts for the development of innovations addressing societal problems. The diversity in Nigeria’s context and high inequality within and between populations should be considered at every stage of development to get feedback from different populations. In addition, it is important to combine mobile health technologies to address a particular problem such as mobile applications together with social media, SMS, calls, or websites. Finally, there is a need for large-scale studies and longitudinal follow-up to understand the efficiency of the mobile application and inform iteration to address specific problems affecting pregnant women in Nigeria.

## Conclusion

mHealth applications have the potential to transform healthcare delivery in sub-Saharan Africa by providing cost-effective alternatives to the traditional method of care. However, there is still limited evidence and framework guiding the development of quality, effective, and sustainable mHealth applications. This study has described the process, challenges, and lessons learned in the development of the first indigenous mobile application specifically for pregnant women in Nigeria and other developing countries using a user-centered design thinking approach. Future research should focus on a pilot study to evaluate the usability and efficiency of the MyBelle pregnancy application in reducing pregnancy deaths and complications among users using a randomized controlled trial.

## Data Availability

The raw data supporting the conclusions of this article will be made available by the authors, without undue reservation.
